# The effects of an integrated care model on frail elderly

**Published:** 2012-09-04

**Authors:** W.M Looman, I.N Fabbricotti

**Affiliations:** Erasmus University Rotterdam, The Netherlands; Erasmus University Rotterdam, The Netherlands

**Keywords:** integrated care, frail elderly, effects

## Abstract

Organisation of health care for frail elderly is no longer adequate. Their complex requirements within the domains of prevention, care, cure, welfare and residence demand a new care model. Partitions need to be broken down and care-providers should work together on a single, integral basis, from diagnosis to treatment. This type of integrated care model has recently been implemented in the region of Walcheren in the Netherlands.

Frail elderly living at home are preventively detected with the Groningen Frailty Indicator and their care demands are assessed with the EASYcare-instrument. Geriatric nurse practitioners and secondary care geriatric nursing specialists become supervisors of the care plans and care coordinators, whereas the G.P. is appointed as director and partner in prevention and care. The entire process is encompassed in multidisciplinary protocols, files (web-based), and multidisciplinary meetings and consultation within and between primary, secondary and tertiary care.

The present study evaluates this integrated care model by examining its effects on frail elderly. Effects of the integrated care model on frail elderly are assessed for ADL-functions, experienced health, mental well-being, social functioning, quality of life and satisfaction with the provision of care. Furthermore, the effect on the use of care is explored. Validated instruments, such as the Katz-15 and Rand-36 are used.

The design of this study is quasi-experimental: approximately 200 frail elderly patients of six G.P. practices that have implemented the integrated care model are compared with 200 frail elderly patients from six G.P. practices that have provided regular care. For this study, baseline measures will be compared with a three-month and a one-year follow-up. At the conference, the results of the three-month follow-up will be presented.

Powerpoint presentation available at http://www.integratedcare.org at congresses – San Marino – programme.

